# Distinct circulating cytokine/chemokine profiles correlate with clinical benefit of immune checkpoint inhibitor monotherapy and combination therapy in advanced non‐small cell lung cancer

**DOI:** 10.1002/cam4.5918

**Published:** 2023-04-16

**Authors:** Yue Hu, Shixun Li, He Xiao, Yanli Xiong, Xianfeng Lu, Xiao Yang, Wei Luo, Jiamin Luo, Shiheng Zhang, Yi Cheng, Lei Zhang, Xiaoyan Dai, Yuxin Yang, Dong Wang, Mengxia Li

**Affiliations:** ^1^ Cancer Center, Daping Hospital Army Medical University Chongqing China

**Keywords:** biomarkers, chemokines, cytokines, non‐small cell lung cancer, PD‐1/PD‐L1

## Abstract

**Background:**

An ever‐increasing number of efforts are focused on identifying effective biomarkers for immune checkpoint inhibitors (ICIs). Cytokines and chemokines are critical to tumor growth, metastasis, tumor angiogenesis, and the immune response against tumor cells. In the study here, we determined the correlation between circulating cytokines/chemokines and the clinical benefit of ICIs for non‐small cell lung cancer (NSCLC) patients.

**Methods:**

Peripheral blood samples were collected before and during treatment (at 12th week). Plasma levels of cytokines/chemokines and specific stress response markers were measured using the Bio‐Plex Pro Human Cytokines Grp I Panel (27‐plex), an APEX1 detection kit, and a human LAP(TGF‐β1) immunoassay kit. A Mann–Whitney *U*‐test or Wilcoxon signed‐rank test and a Cox proportional hazards model were employed for statistical analysis.

**Results:**

In the ICI monotherapy cohort, a high level of IL‐6 at pretreatment or an elevation of IL‐6, IL‐8, FGF2, CXCL10, CCR1, PDFGB, TNF, and APEX1 posttreatment was associated with poor progress‐free survival (PFS). A posttreatment elevation (defined herein as change rate) of CXCL10 was also associated with poor overall survival (OS). In the combinational therapy group, a high level of IL‐12, IL‐17A, FGF2, VEGF, and APEX1 at pretreatment and an elevation of CCL2 posttreatment were associated with poor PFS. A high level of IL‐9, FGF2, PDFGB, CCL4, TFGB, and APEX1 at pretreatment and an elevation of IL‐13, CSF2, and CCL2 at posttreatment were associated with poor OS of patients receiving combination therapy.

**Conclusions:**

The study here suggests that circulating cytokines/chemokines are feasible, noninvasive biomarkers for predicting clinical benefit of ICI treatment for NSCLC. Distinct circulating factor profiles were observed in individuals receiving ICI monotherapy or combination therapy.

## 1 INTRODUCTION

With considerable morbidity and mortality, lung cancer ranks as a leading cause of death among all malignancies.^1^ More than 85% of patients are initially diagnosed with advanced stage non‐small cell lung cancer (NSCLC), and the 5‐year survival rate is only 16%.^2^ Although a better prognosis can be attained with targeted therapy in patients harboring a subset of driver gene alterations, individualized therapy is actionable on only a limited number of driver genes, while acquired resistance is inevitable in most patients.^3^, ^4^, ^5^

Immune checkpoint inhibitors (ICIs), which utilize PD‐1/PD‐L1 or CTLA‐4 antibodies to block key checkpoint regulators, have been widely used in NSCLC patients in the clinic.^6^ Patients who respond to the first couple of doses of ICIs are expected to maintain tumor growth suppression and live longer than those who experience tumor progression in the first 6 months.^7^ Recently, the use of ICIs in neoadjuvant therapies has revealed dramatic tumor regression in surgical tissues, providing a likely explanation for the survival benefit of ICIs in NSCLC patients.^8^, ^9^ Unfortunately, the proportion of NSCLC patients who effectively respond to ICIs is relatively limited.^10^ Many efforts have been put forth to increase the response rate to ICIs, such as combining ICIs with chemotherapy, radiotherapy, or other systemic treatments. However, additional toxicities introduced by combination therapy are common, compromising the sustainable clinical benefit of the immunotherapy.^11^, ^12^, ^13^

Inspired by the success of targeted therapies in the precision medicine era, biomarkers expected to predict efficacy of ICIs have been searched for in patients who respond to ICIs. The expression of the PD‐L1 molecule in tumor cells or the tumor microenvironment (TME) is the first established biomarker for ICIs. Indeed, PD‐L1 levels predict the response rate, as well as the progress‐free survival (PFS) or overall survival (OS) benefit, for specific PD‐1 monoclonal antibodies, alone or with chemotherapy, in treating NSCLC.^14^ As such, PD‐L1 assessment using immunohistochemistry (IHC) has now been approved by the US Food and Drug Administration (FDA) and other agencies as a companion diagnostic test. However, considering the heterogeneity of PD‐L1 expression in the TME and the considerable variation among PD‐L1 assay kits, the application of this biomarker in clinical practice is still complicated.^15^ Tumor mutational burden (TMB) was considered a promising pan‐cancer predictive biomarker due to its correlation with immunogenicity of tumor cells.^16^, ^17^ However, the clinical application of TMB has been held back due to the lack of supporting data from prospective clinical trials, the lack of a standardized assay, and the lack of predictive efficacy for combination ICI therapies.^18^, ^19^ Presently, there are quite a few potential biomarkers under investigation, yet they each have long paths forward involving clinical trials.

Among the biomarkers under development for ICIs, ones from peripheral blood have unique advantages mainly due to their minimally noninvasive and routine collection in clinical practice. Peripheral blood provides a source to analyze tumor cells and the TME and to monitor global and specific responses to ICIs. Cytokines and chemokines are widely known as two families of small soluble proteins that have intricately intercellular signaling function both in innate and adaptive immune reactions.^20^, ^21^, ^22^, ^23^ Cytokines and chemokines secreted by tumor cells, tumor‐infiltrating immune cells, and tumor stromal cells created an intricate local immune microenvironment and affected systemic immune response.^24^, ^25^ Among cytokines and chemokines, IL‐1β, IL‐6, IL‐8, IL‐12, and TNF‐α were already known as pro‐inflammation factors, whereas IL‐4 and IL‐1RA were known as anti‐inflammatory factors in immune response both physiologically and pathologically.^23^ It has been proved that the composition of TME can be influenced by the inflammation and the progression of NSCLC.^26^, ^27^ Previous studies have reported that some chemokines and cytokines, such as IFN‐γ and IL‐18, in peripheral blood are associated with responsiveness to ICIs in NSCLC, suggesting that they are candidate ICI biomarkers.^28^, ^29^ However, those studies failed to comprehensively determine the profile of circulating factors in peripheral blood. In addition, due to the limitation of the enrolled patient cohort, differences in circulating factor profiles in mono or combination therapies involving ICIs has yet to be examined. Thus, the value of peripheral bioactive molecules in predicting ICI efficacy requires further investigation.

In this study, we aimed to discover feasible circulating biomarkers of ICI efficacy and safety in NSCLC patients receiving ICIs. Toward that goal, a Bio‐Plex MAGPIX Multiplex System was employed to determine the profile of 27 plasma cytokines/chemokines in NSCLC patients who received treatment with PD‐1/PD‐L1 inhibitors alone or in combination with chemotherapy. In addition, a DNA repair, stress response protein, apurinic/apyrimidinic endonuclease (APEX1), was included in current study, as previous reports showed it can be detected in peripheral blood samples^30^ and its serum level is associated with platinum containing chemotherapy efficacy in NSCLC patients.^31^

## 2 METHODS

### 2.1 Patients and study design

From 2018 to 2019, 51 patients enrolled in the study. Patients were histologically confirmed to possess unresectable stage III‐IV advanced NSCLC. Patients with any of the following conditions were excluded: adenocarcinoma with actionable mutation (e.g., EGFR, ALK, ROS1, RET); severe immune‐related diseases; history of hematopoietic stem cell or organ transplantation; Eastern Cooperative Oncology Group performance status (ECOG‐PS) >2, or pregnant. The study was conducted using two cohorts: monotherapy and combination therapy. In the monotherapy cohort, patients received PD‐1/PD‐L1 inhibitors only: nivolumab 240 mg for 2 weeks, pembrolizumab 2 mg/kg for 3 weeks, sintilimab 200 mg for 3 weeks, toripalimab 3 mg/kg for 3 weeks or atezolizumab 1200 mg for 3 weeks. In the combination cohort, patients were treated with PD‐1/PD‐L1 inhibitors plus chemotherapy involving platinum and taxol/gemcitabine/pemetrexed. The study protocol was approved by the Institutional Ethics Board of Daping Hospital. Written informed consent was obtained from all patients for use of blood samples and clinical information.

### 2.2 Sample collection

Peripheral blood samples were obtained by venipuncture at baseline (prior to therapy, pretreatment) and the time of second clinical evaluation (at 12 weeks, posttreatment). After collection, blood samples were centrifuged (1000 × g, 10 min) to isolate plasma. Plasma samples were stored at −80°C until laboratory analysis. No freeze–thaw cycles were allowed before analysis.

### 2.3 Cytokines and chemokines assay

Plasma levels of 27 cytokines/chemokines were measured using the Bio‐Plex Pro Human Cytokines Grp I Panel 27‐plex according to the manufacturer's instructions. In brief, plasma samples were incubated in 96‐well plates embedded with microbeads for 30 min, then incubated with detection antibody for 30 min. Afterwards, streptavidin‐phycoerythrin (SA‐PE) was added into each well for 10 min. Cytokine values were determined using the Bio‐Plex MAGPIX System, a dual‐laser flow cytometry technology (H‐Wayne Biotechnologies). A total of 51 plasma samples were divided into two batches for measurement. Of the 27 cytokines/chemokines determined, 4 cytokines/chemokines returned negative readouts. The remaining 23 cytokines/chemokines examined in this study were as follows: IL‐1B, IL‐1R1, IL‐2, IL‐4, IL‐6, IL‐7, IL‐8, IL‐9, IL‐12, IL‐13, IL‐17A, CCL11, FGF2, CSF3, CSF2, CXCL10, CCL2, CCR1, PDGFB, CCL4, CCL5, TNF, and VEGF.

### 2.4 APEX1 detection kit

The quantitative determination of APEX1 was measured using the Human DNA‐(Apurinic or Apyrimidinic Site) Lyase (APEX1) ELISA Kit (CUSABIO, Catalog Number CSB‐EL00900HU). Briefly, samples and standards were added to designed wells and incubated for 2 h at 37°C. Liquid was then removed, and the Biotin‐antibody was added and incubated for 1 h at 37°C. Each well was aspirated and washed with wash buffer, repeating the process two times. Horseradish peroxidase (HRP)‐avidin was added and incubated for 1 h at 37°C. Afterwards, the aspiration and wash cycle were repeated five times. Tetramethylbenzidine (TMB) substrate was then incubated for 30 min at 37°C and Stop Solution added. Finally, the optical density of each well was then determined within 5 min using a microplate reader set to 450 nm.

### 2.5 Human LAP (TGF‐β1) immunoassay kit

For the quantitative determination of TGF‐β1, a Quantikine ELISA (Catalog Number DLAP000) was used. According to the instructions, each well was filled accordingly with assay diluent, then either standard, control or samples were added and incubated for 2 h at room temperature on a horizontal orbital microplate shaker setting at 500 rpm. Each well was aspirated and washed with wash buffer four times. Afterwards, the human LAP (TGF‐β1) conjugate was added and incubated for 2 h at room temperature on the shaker, followed by another four washes. The Substrate Solution was then added and incubated for 30 min at room temperature on the benchtop. After the addition of stop solution, the readings at 450 nm were determined using an optical density with correctable function.

### 2.6 Response evaluation and follow‐up

Computed tomography (CT) scans of the chest and abdomen and nuclear magnetic resonance imaging (MRI)/CT scan of the brain were performed at baseline and every 6 weeks after starting treatment. Tumor burden was measured in all patients by radiographic imaging. As Immunotherapy Related Response Evaluation Criteria in Solid Tumors (irRECIST) had not been widely adopted in clinical practice at the time of this study, the responses were assessed according to RECIST v1.1. Patients were categorized into complete response (CR), partial response (PR), stable disease (SD), and progressive disease (PD). Durable clinical benefit (DCB) was defined as patients who were categorized as CR, PR, or SD for ≥24 weeks, and non‐durable clinical benefit (NDB) was defined as patients who were categorized as PD or SD for <24 weeks.^32^ Immune‐related adverse events (irAEs) were monitored and evaluated during treatment. The National Cancer Institute Common Terminology Criteria for Adverse Events version 5.0 (CTCAE v5.0) were utilized to grade irAEs.

### 2.7 Statistical process and analysis

All concentrations of cytokines/chemokines were expressed as median and 25% or 75% percentile values (median, Q1–Q3). The change rate of cytokines/chemokines was defined as the difference between before and after ICI treatment divided by the pretreatment level. The Mann–Whitney *U* test or the Wilcoxon signed‐rank test was applied to assess differences in cytokine/chemokine levels or changes between DCB and NDB patients. Receiver operating characteristic (ROC) curves were used to determine cytokine/chemokine plasma concentrations or change rates to discriminate DCB patients from NDB patients. The Youden index was used to evaluate the optimal cut‐off value(s) to predict response. A univariate and multivariate Cox proportional hazards model was performed to analyze independent prognostic factors for PFS and OS. For the development of progression risk and death risk models based on circulating factors, forward stepwise regression was applied by integrating circulating factors with significance (Ward *p* < 0.1) as candidate variables in univariate Cox regression. *p* < 0.1 and *p* < 0.2 was used as criteria for including and excluding one variable based on the likelihood ratio test. The Kaplan–Meier (K–M) method and log rank test were used to assess differences in PFS and OS. All statistical analysis was carried out with IBM SPSS Statistic 22 (IBM SPSS, Chicago). A two‐tailed *p* < 0.05 value was considered statistically significant.

## 3 RESULTS

### 3.1 Patient characteristics

The median age of all patients (monotherapy and combination therapy) was 63 years (range, 31–83 years). The percentage of males and females was 86.3% and 13.7%, respectively. Thirty‐eight (74.5%) patients were smokers. The number of patients who were histologically diagnosed with adenocarcinoma or squamous cell carcinoma was 22 (43.1%) and 29 (56.6%), respectively. At the time of analysis, the median follow‐up time was 20 months. According to the therapy regimen, 33 (64.7%) patients received ICI monotherapy, while 18 (35.3%) patients received a combination of ICI and chemotherapy. The median PFS and OS for all patients was 9.0 months (95% CI: 4.41–13.6) and 14.0 months (95CI%: 9.1–19.9), respectively. According to RECIST v1.1, the number of patients evaluated as CR, PR, SD, or PD was 1 (2.0%), 19 (37.3%), 13 (25.5%), and 18 (35.3%), respectively. Patients were then categorized as DCB (28, 54.9%) or NDB (23,45.1%) according to the criteria described Section 2 and elsewhere.^33^, ^34^, ^35^ The clinical features have been summarized in Table 1.

**TABLE 1:** Clinical characteristic of whole population.

		*n* (%)
Sex	Female	7 (13.7)
	Male	44 (86.3)
Smoking status	Never smoker	13 (25.5)
	Current or former smoker	38 (74.5)
Histology	Adenocarcinoma	22 (43.1)
	Squamous carcinoma	29 (56.9)
Treatment line	First	21 (41.2)
	Second	22 (43.1)
	Third/fourth	8 (15.7)
ICI regimen	ICI monotherapy	33 (64.7)
	ICI plus chemotherapy	18 (35.3)
Clinical benefit	NDB	23 (45.1)
	DCB	28 (54.9)
Response	Non‐response	31 (60.8)
	Response	20 (39.2)
PFS	Censored	17 (33.3)
	Event	34 (66.7)
OS	Censored	22 (43.1)
	Event	29 (56.9)

Abbreviations: DCB, durable clinical benefit; ICI, immune checkpoint inhibitor; NDB, non‐durable clinical benefit.

### 3.2 Association between cytokine/chemokine levels and tumor response of whole cohort

**FIGURE 1:**
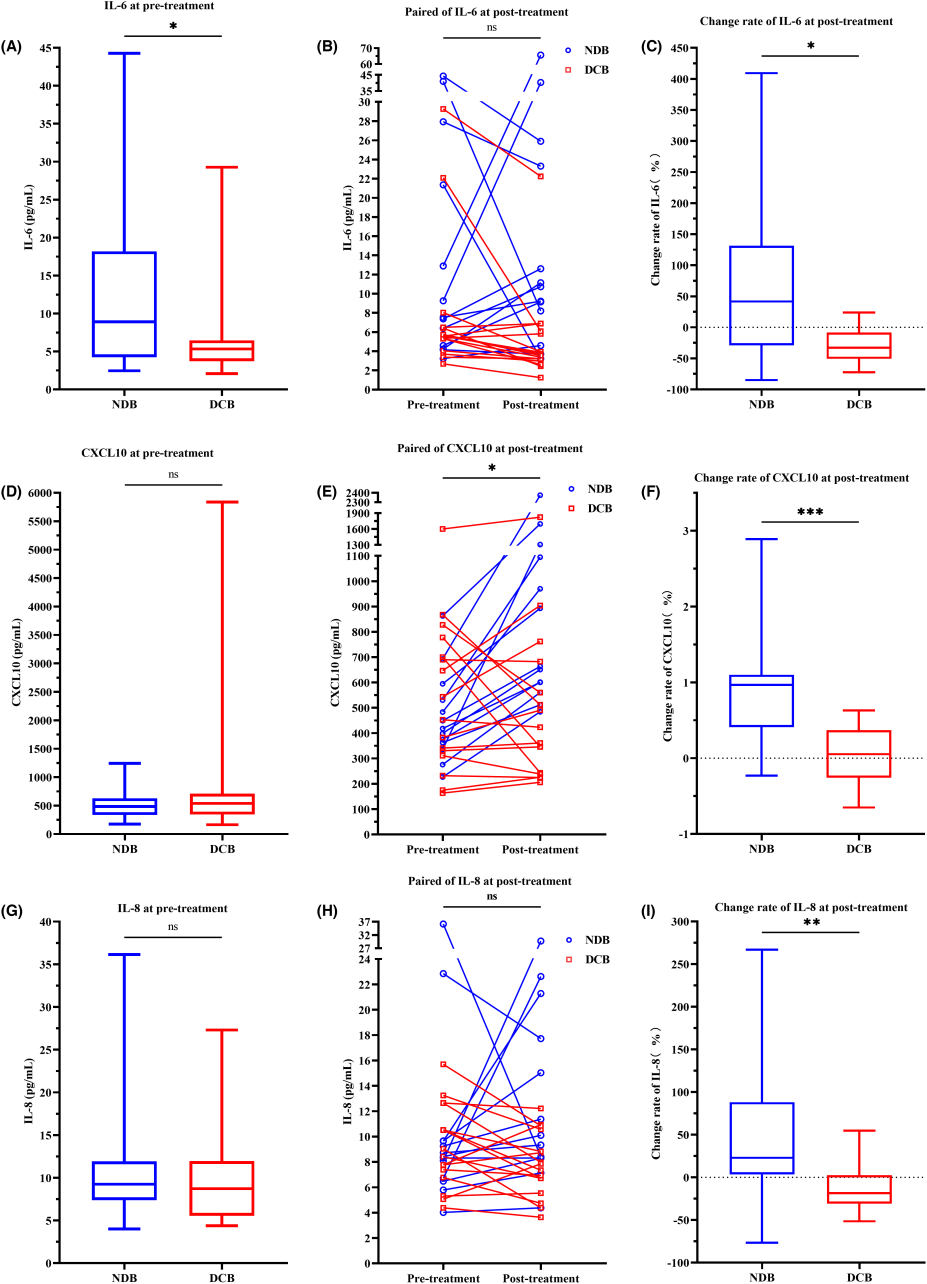
Comparison of IL‐6, CXCL 10 and IL‐8 in the full cohort. (A, D, G). Box plot showing difference in pretreatment level between NDB and DCB patients. (B, E, H). Line series showing dynamics before and after ICI treatment. (C, F, I) Box plot showing difference of change rates between DCB and NDB patients. *p < 0.05, **p < 0.01, ***p < 0.001; ns stands for non‐significance.

Of the 27 cytokines/chemokines tested using the 27‐Plex platform (see Section 2), 4 cytokines/chemokines returned negative readouts, possibly due to insufficient expression in NSCLC. Thus, including APEX1 and TGF‐β, a total number of 25 circulating bioactive factors (see additional list in Section 2) were successfully measured in the pretreatment plasma samples of the 51 study participants (statistical analysis shown in Table 2). We first assessed the relationship between cytokine/chemokine levels and the clinical response of all patients. At pretreatment, IL‐4 was significantly higher in DCB patients than in NDB patients (median (IQR): 2.94 (2.68–3.20) pg/mL vs. 2.59 (2.40–2.93) pg/mL, *p* = 0.043). Compared with NDB patients, only the IL‐6 level at pretreatment was found significantly decreased in DCB patients (median (IQR): 5.45 (3.71–7.64) pg/mL vs. 8.90(4.23–18.19) pg/mL, *p* = 0.049) (Figure 1A,D,G).

**TABLE 2:** Baseline levels of plasma cytokine/chemokine in whole population and difference in clinical benefit subgroups.

	Whole population	NDB^a^ (*n* = 23)	DCB^a^ (*n* = 28)	*p*
	Median (Q1, Q3) (pg/mL)	Median (Q1, Q3) (pg/mL)	Median (Q1, Q3) (pg/mL)	
IL‐1B	2.51 (2.07–3.23)	2.40 (2.08–3.23)	2.58 (1.97–4.09)	0.798
IL‐1R1	320.13 (255.43–444.59)	306.78 (237.00–557.29)	331.98 (270.36–376.56)	0.872
IL‐2	5.81 (4.39–7.36)	6.33 (5.28–8.17)	5.35 (3.94–7.30)	0.103
IL‐4	2.81 (2.53–3.13)	2.59 (2.40–2.93)	2.94 (2.68–3.20)	0.043
IL‐6	6.26 (3.95–13.02)	8.90 (4.23–18.19)	5.45 (3.71–7.64)	0.049
IL‐7	29.10 (22.35–34.97)	23.47 (20.35–34.13)	29.38 (25.99–35.41)	0.100
IL‐8	9.04 (6.48–11.92)	9.25 (7.39–11.92)	8.72 (5.56–11.96)	0.478
IL‐9	63.40 (55.60–68.86)	63.922 (56.72–70.53)	60.32 (51.20–68.44)	0.205
IL‐12	4.5 (3.59–5.47)	4.73 (3.93–5.47)	4.24 (3.25–5.96)	0.348
IL‐13	7.96 (6.22–11.27)	7.11 (5.56–8.21)	8.83 (6.45–13.11)	0.083
IL‐17A	8.48 (7.49–9.96)	8.54 (7.85–9.96)	8.48 (7.29–9.98)	0.525
CCL11	51.08 (34.69–66.49)	43.60 (31.77–62.53)	57.86 (41.40–75.95)	0.078
FGF2	30.00 (25.88–36.29)	32.14 (26.33–36.29)	29.01 (24.86–37.19)	0.410
CSF3	75.52 (52.32–94.73)	78.02 (55.15–114.56)	73.05 (51.06–77.99)	0.156
CSF2	2.18 (1.16–3.83)	1.16 (1.16–3.75)	2.95 (1.16–5.03)	0.146
CXCL10	511.00 (341.20–692.83)	484.28 (336.04–629.78)	539.57 (343.18–710.96)	0.508
CCL2	11.06 (6.25–17.16)	11.06 (6.08–17.55)	11.47 (6.67–16.76)	0.977
CCR1	3.29 (2.79–4.32)	3.65 (2.92–5.83)	3.22 (2.70–3.97)	0.233
PDGFB	726.00 (475.19–1235.95)	697.72 (554.11–1185.19)	731.53 (386.83–1253.20)	0.719
CCL4	48.49 (43.28–54.75)	50.12 (47.09–55.02)	45.77 (42.00–54.20)	0.100
CCL5	3806.82 (3099.31–5093.59)	4081.79 (2909.76–4929.68)	3804.14 (3127.06–5469.00)	0.733
TNF	29.92 (26.75–35.62)	31.23 (27.09–35.68)	28.38 (24.06–34.60)	0.233
VEGF	78.66 (48.01–107.35)	87.91 (48.01–125.80)	75.66 (50.00–106.50)	0.616
TGFB1	21.61 (11.09–30.57)	22.90 (15.45–30.57)	15.37 (8.89–30.91)	0.252
APEX1	90.75 (27.63–131.38)	104.50 (43.88–148.25)	65.75 (16.69–115.13)	0.106

Abbreviations: DCB, durable clinical benefit; NDB, non‐durable clinical benefit.

^a^ Categorize criteria see in response evaluation and follow up of Section 2.

Twenty‐nine paired plasma samples were collected at pretreatment and at 12 weeks post the first dose of PD‐1/PD‐L1 inhibitors. IL‐4 (*p* = 0.018), IL‐12 (*p* = 0.042), IL‐17A (*p* = 0.042), CFS3 (*p* = 0.020), TNF (*p* = 0.026), CCR1 (*p* = 0.024) (Table S1, Figure S1), and CXCL10 (*p* = 0.014) (Figure 1B,E,H) were each found to be significantly higher in posttreatment samples compared to pretreatment samples. Exploring further the association between the clinical response and the dynamic change rate of plasma bioactive factors uncovered that several factors were significantly increased in NDB patients relative to DCB patients: IL‐6 (*p* = 0.022), CXCL10 (*p* < 0.001), IL‐8 (*p* = 0.008) (Figure 1E,F,I), CCL2 (*p* = 0.025), CCR1 (*p* = 0.015), and CCL5 (*p* = 0.028) (Figure S2). This finding indicates that the change rates of these six bioactive factors are more effective at identifying the responders than the posttreatment level.

**FIGURE 2:**
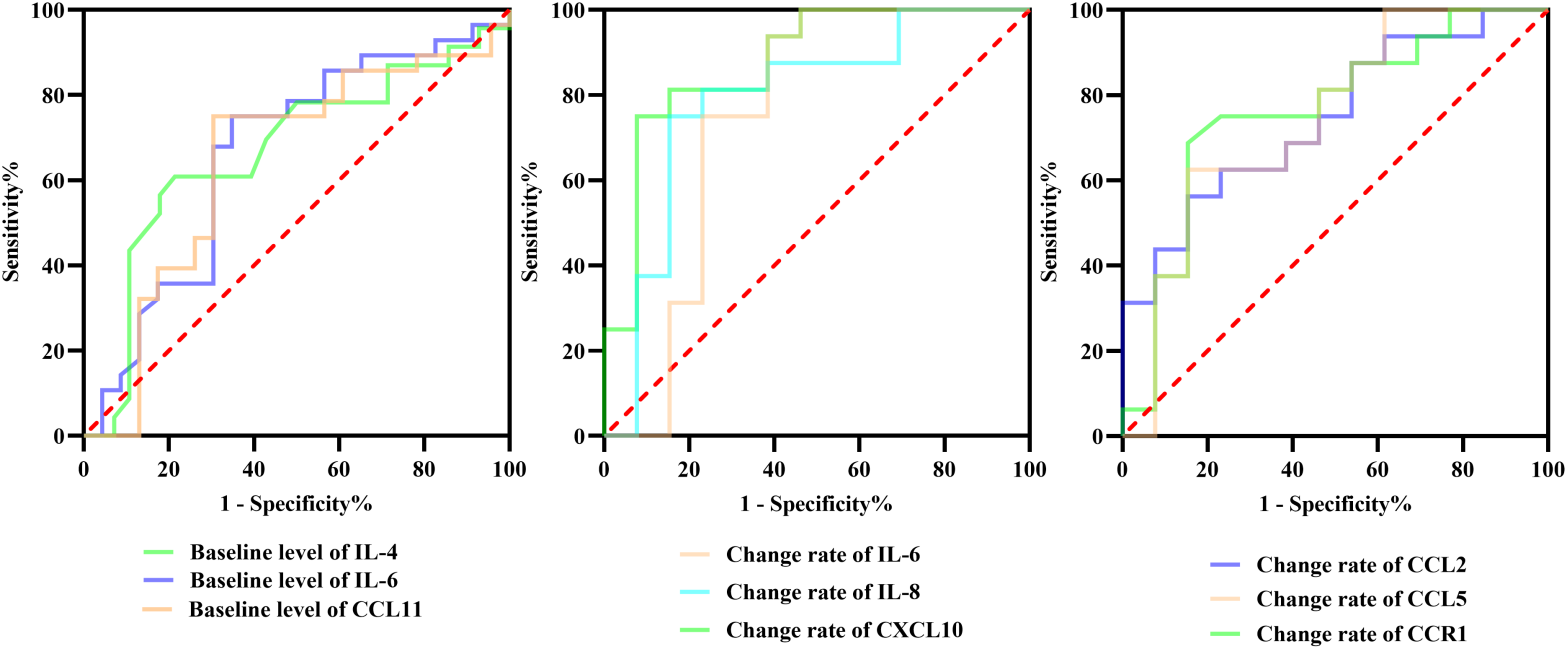
Area under curve of receiver operating characteristic curve analysis for IL‐4, IL‐6, or CCL11 level at pretreatment, change rate of IL‐6, IL‐8, or CXCL10, and change rate of CCL2, CCL5 and CCR1 at posttreatment.

IL‐4, IL‐6, and CCL11 at pretreatment and the change rate of IL‐6, IL‐8, CXCL10, CCL2, CCL5, and CCR1 exhibited varying capacities to discriminate DCB from NDB patients. The area under curve (AUC) for each cytokine/chemokine at pretreatment or following treatment are shown in Table 3 and Figure 2. Based on the Youden index, four cut‐off values were identified for the pretreatment level: IL‐4 > 2.65 pg/mL, IL‐6 > 6.93 pg/mL, CCL11 > 47.05 pg/mL; or the change rate: CXCL10 > 28.47%, IL‐6 > 69.36%, IL‐8 < −1.69%, CCL2 < 4.91%, CCL5 > ‐0.16%, and CCR1 > 8.48%.

**TABLE 3:** Area under curve from the analysis of receiver operating characteristic curve of baseline or change rates of several cytokines/chemokines for predicting clinical benefit.

	AUC (95% CI)	*p*
Baseline level of IL‐4	0.666 (0.509–0.823)	0.043
Baseline level of IL‐6	0.668 (0.458–0.879)	0.125
Baseline level of CCL11	0.664 (0.484–0.805)	0.078
Change rate of IL‐6	0.750 (0.541–0.959)	0.023
Change rate IL‐8	0.788 (0.607–0.970)	0.009
Change rate of CXCL10	0.875 (0.741–1.000)	0.001
Change rate of CCL2	0.745 (0.567–0.923)	0.025
change rate of CCL5	0.740 (0.551–0.930)	0.028
Change rate of CCR1	0.764 (0.581–0.948)	0.016

#### 3.2.1 Association between plasma cytokine/chemokine levels and PFS of whole cohort

Univariate Cox regression revealed that there is no significant association between PFS and pretreatment levels of the tested cytokines/chemokines (Table S3). During the course of cancer therapy, a high change rate of IL‐6, IL‐8, CXCL10, CCR1, and CCL5 was significantly associated with poor PFS (Table S4). However, multivariate Cox regression showed that there was no independent risk factor for PFS among the cytokine/chemokine levels or the clinical characteristics.

**FIGURE 3:**
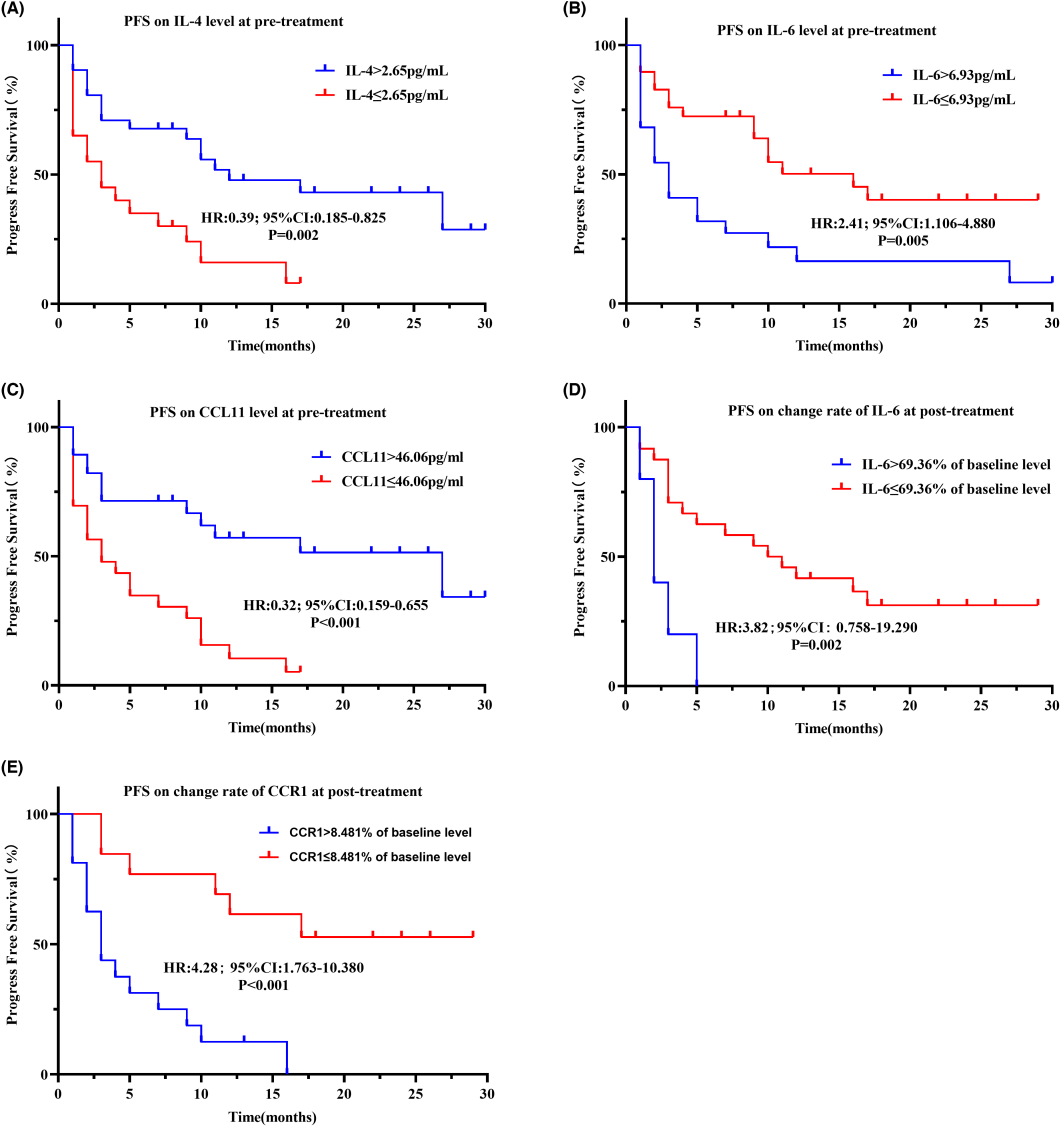
Kaplan–Meier curves illustrating differences in prognosis in the full cohort. PFS based on IL‐4 level at pretreatment (A), IL‐6 level at pretreatment (B), CCL11 level at pretreatment (C), change rate of IL‐6 (D), change rate of CCR1 (E).

Additionally, a K–M curve was generated using the cut‐off values determined by the ROC analysis above. It was found that PFS was significantly shorter with a high level of IL‐6 at pretreatment (high vs. low: HR = 2.41, 95% CI: 1.106–4.880, log rank *p* = 0.005), a high change rate of IL‐6 at posttreatment (high vs. low: HR = 3.82, 95CI%: 0.758–19.290, long rank *p* = 0.002), or a high change rate of CCR1 at posttreatment (high vs. low: HR = 4.28, 95CI%: 1.763–10.380, log rank *p* < 0.001). Conversely, a longer PFS was significantly associated with a high level of IL‐4 at pretreatment (high vs. low: HR = 0.39, 95CI%: 0.185–0.825, log rank *p* = 0.002) and high level of CCL11 at pretreatment (high vs. low: HR = 0.32, 95% CI: 0.159–0.655, log rank *p* < 0.001) (Figure 3, Table 4).

**TABLE 4:** Progress‐free survival time hazard ration of whole cohort.

	HR	95% CI	*p*
IL‐4 at pretreatment (high vs. low)	0.39	0.185–0.825	0.002
IL‐6 at pretreatment (high vs. low)	2.41	1.106–4.880	0.005
CCL11 at pretreatment (high vs. low)	0.32	0.158–0.655	<0.001
Change rate of IL‐6 (high vs. low)	3.82	0.758–19.290	0.002
Change rate of CCR1 (high vs. low)	4.28	1.763–10.380	<0.001

**FIGURE 4:**
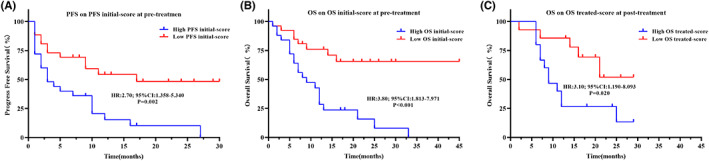
Kaplan–Meier curves illustrating difference in prognosis in the whole cohort using prognostic models. (A) PFS based on PFS initial‐score at pretreatment. (B) OS based on OS initial‐score at pretreatment. (C) OS based on OS treated‐score at posttreatment.

To confirm a role of circulating factors in prognosis independent of clinical factors, such as DCB, linear weighted models were developed using forward stepwise Cox regression. The formula for progression risk using pretreatment levels of the circulating factors is (PFS initial‐score): 0.061×baseline level of IL8–0.019×baseline level of CCL11. Patients were divided into two groups, that is high progression risk and low progression risk, by the median value of the PFS initial‐score. In contrast to the weak association of one pretreatment circulating factor with the PFS (Tables S3 and S4), the PFS initial‐score was strongly, significantly associated with PFS (Table 5), where a high PFS initial‐score associated with poor PFS (high vs. low: HR:2.70; 95% CI: 1.358–5.340; *p* = 0.002) (Figure 4A). Importantly, the PFS initial‐score remained significant after adjusted for clinical response. At posttreatment, only the change rate of IL‐6 was significantly different when a forward stepwise was performed. After adjusted for DCB and response, only the change rate of IL‐6 was significant for PFS in treated models (Table 5).

**TABLE 5:** Prognostic models developed from the combination of several circulating factors.

Initial models (*n* = 51)	Univariate Cox		Multivariate Cox^a^		Multivariate Cox^b^	
	HR (95% CI)	*p*	HR (95% CI)	*p*	HR (95% CI)	*p*
PFS score (continuous)	2.72 (1.356–5.449)	0.005	2.17 (0.923–5.112)	0.076	2.67 (1.282–5.553)	0.009
PFS score (categorized high vs. low)	2.77 (1.358–5.665)	0.005	1.30 (0.591–2.848)	0.517	2.55 (1.239–5.249)	0.011
OS score (continuous)	2.72 (1.556–4.745)	<0.001	2.47 (1.306–4.653)	0.005	3.40 (1.860–6.220)	<0.001
OS score (categorized high vs. low)	3.92 (1.722–8.930)	0.001	2.06 (0.851–4.979)	0.109	5.29 (2.154–12.988)	<0.001

Treated models (*n* = 29)	Univariate Cox		Multivariate Cox^a^		Multivariate Cox^b^	
	HR (95% CI)	*p*	HR (95% CI)	*p*	HR (95% CI)	*p*
PFS score^c^ (continuous)	1.849 (1.242–2.752)	0.002	1.272 (0.821–1.970)	0.282	1.449 (0.938–2.238)	0.094
PFS score^c^ (categorized high vs. low)	4.420 (1.672–11.685)	0.003	1.250 (0.424–3.685)	0.685	1.452 (0.478–4.404)	0.510
OS score (continuous)	2.72 (1.451–5.091)	0.002	1.43 (0.685–2.989)	0.341	1.87 (0.938–3.717)	0.075
OS score (categorized high vs. low)	3.21 (1.115–9.241)	0.031	1.61 (0.491–5.294)	0.431	2.77 (0.883–8.711)	0.081

Abbreviations: OS, overall survival; PFS, progress‐free survival.

^a^ Adjusted for DCB.

^b^ Adjusted for response.

^c^ Only change rate of IL‐6 was in this model and categorized.

#### 3.2.2 Association between plasma cytokine/chemokine levels and OS of whole cohort

Univariate Cox regression showed that IL‐1R1 and IL‐6 levels at pretreatment were significantly associated with poor OS. A high pretreatment level of CCL11 was significantly associated with a better OS (Table S5), whereas a high change rate of IL‐6, CXCL10, and CCL2 were significantly associated with poor OS (Table S6). Nonetheless, the pretreatment level of IL‐1R1 is the only independent prognostic marker for OS with a 9.3% increase in death risk per 100 pg/mL change rate for the receptor (Table 6).

**TABLE 6:** Multivariate Cox regression for overall survival at pretreatment level.

	*β*	SE	Wald	*p*	HR (95% CI)
DCB (DCB vs. NCB)	−3.463	0.671	25.161	0.000	0.035 (0.009–0.129)
IL‐1R1	0.001	0.000	8.501	0.004	1.093 (1.030–1.161)^a^
IL‐6	0.017	0.017	1.005	0.316	1.017 (0.984–1.0519)
CCL11	−0.019	0.010	3.413	0.065	0.981 (0.961–1.001)

^a^ The HR and corresponding 95% confidential interval was calculated for increasing in/100 units.

**FIGURE 5:**
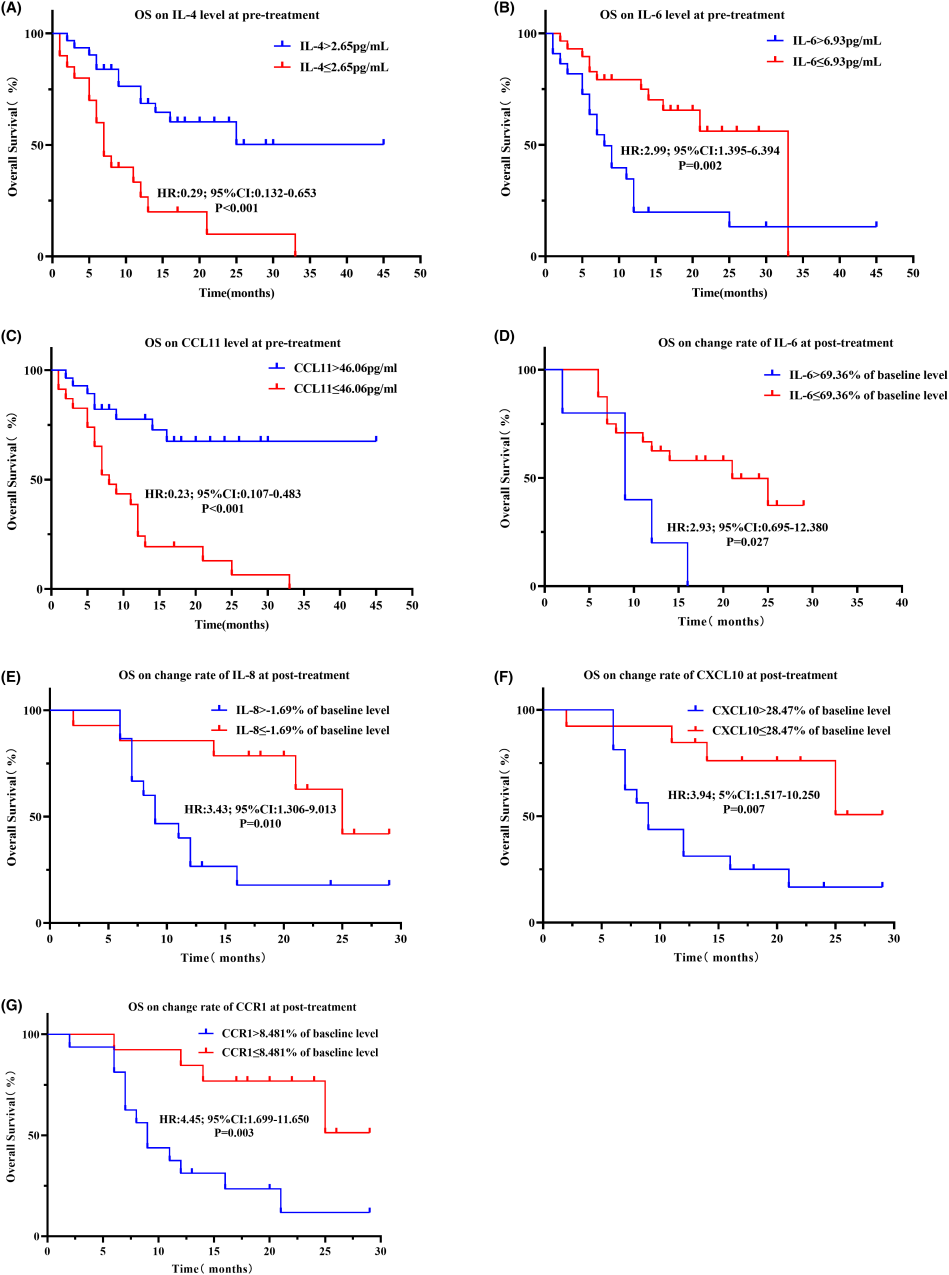
Kaplan–Meier curves illustrating difference in prognosis in the full cohort. OS based on IL‐4 level at pretreatment (A), IL‐6 level at pretreatment (B), CCL11 level at pretreatment (C), change rate of IL‐6 (D), change rate of IL‐8 (E), change rate of CXCL10 (F), or change rate of CCR1 (G).

Using the same cut‐off points, the K–M curves showed that a low pretreatment level of IL‐4 (high vs. low: HR:0.29, 95% CI: 0.132–0.653, log rank *p <* 0.001), a high pretreatment level of IL‐6 (high vs. low: HR:2.99, 95% CI: 1.395–6.394, log rank *p* = 0.002), a low pretreatment level of CCL11(high vs. low: HR:0.23, 95% CI: 0.107–0.483, log rank *p <* 0.001), a high change rate in IL‐6 (high vs. low: HR:2.93, 95% CI: 0.695–12.380, log rank *p* = 0.027), a high change rate in IL‐8 (high vs. low: HR:3.43, 95CI%:1.306–9.013, log rank *p* = 0.010), a high change rate in CXCL10 (high vs. low: HR:3.94, 95% CI: 1.517–10.250, log rank *p* = 0.007), or a high change rate in CCR1 (high vs. low: HR:4.445, 95% CI: 1.699–11.650, log rank *p* = 0.003) was significantly associated with poor OS (Figure 5, Table 7). The OS initial model and OS treated‐score model were also converted to an OS initial‐score = 0.001 × pretreatment level of IL1R1–0.026 × pretreatment level of CCL11 and an OS treatment‐score = 0.147 × change rate of CSF2 + 0.838 × change rate of CXCL10. Patients with a high OS initial‐score had a shorter OS (high vs. low: HR:3.80; 95% CI: 1.813–7.971; *p* < 0.001), and a high OS treated‐score was associated with shorter OS (high vs. low:HR:3.10; 95% CI: 1.190–8.093; *p* = 0.020) (Figure 4B,C). Moreover, the OS baseline‐score model, when employed as a continuous variate, was an independent prognostic factor for OS when adjusted for DCB and clinical response (Table 5). Taken together, our results indicate that (i) assessment of pretreatment levels of specific circulating factors and (ii) monitoring of their alterations during ICI treatment are important steps for evaluating and predicting the clinical benefit of this treatment strategy.

**TABLE 7:** Overall survival time hazard ration of whole cohort.

	HR	95% CI	*p*
IL‐4 at pretreatment (high vs. low)	0.29	0.132–0.653	<0.001
IL‐6 at pretreatment (high vs. low)	2.99	0.965–12.380	0.002
CCL11 at pretreatment (high vs. low)	0.23	0.107–0.483	<0.001
Change rate of IL‐6 (high vs. low)	2.93	0.695–12.380	0.002
Change rate of IL‐8 (high vs. low)	3.43	1.306–9.013	0.010
Change rate of CXCL10 (high vs. low)	3.94	1.517–10.250	0.007
Change rate of CCR1 (high vs. low)	4.45	1.699–11.650	0.003

### 3.3 Subgroup analysis of correlation between plasma cytokine/chemokine levels and ICI efficacy

#### 3.3.1 ICI monotherapy cohort

##### 3.3.1.1 Characteristics of ICI monotherapy cohort

**FIGURE 6:**
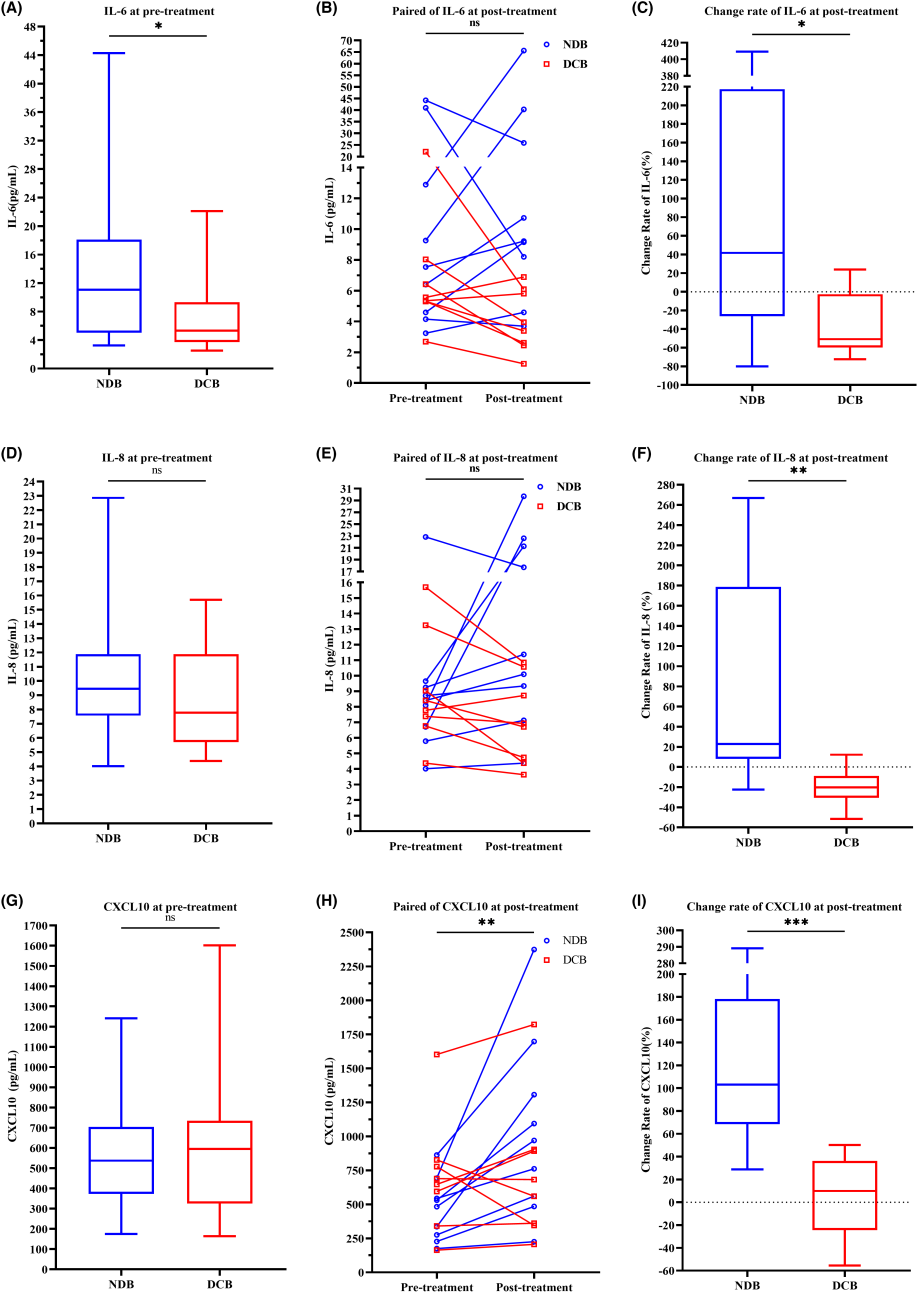
Comparison of IL‐6, IL‐8 and CXCL 10 in the subset of patients that received ICI monotherapy. (A, D, G). Box plot showing differences in baseline levels between NDB and DCB patients. (B, E, H). Line series showing dynamics before and after ICI treatment. (C, F, I) Box plot showing difference of change rate between DCB and NDB patients. * *p* < 0.05, ** *p* < 0.01, *** *p* < 0.001; ns stands for non‐significant.

In our study, there were 33 patients that received monotherapy of PD‐1/PD‐L1 inhibitors. The median age of this cohort was 65 years (range, 46–79 years). The number of male and female was 30 (90.9%) and 3 (9.1%). Twenty‐six (78.8%) patients were smokers. Among them, 9 (27.3%) and 24 (72.7%) were diagnosed with adenocarcinoma or squamous cell carcinoma. At the time of analysis, the median PFS and OS was 7.0 months (95% CI: 6.06–12.42) and 9.0 months (95CI%:9.73–16.94). All of the participants had plasma samples collected before ICI treatment, and 17 paired plasma samples were collected at 12 weeks posttreatment. According to RECIST1.1, there were 1 (3.0%), 10 (30.3%), 8 (24.3%), and 14 (42.4%) participants classified as CR, PR, SD, and PD, respectively. Among the DCB patients, the pretreatment level of IL‐6 was significantly lower than in the NDB patients (median (IQR): 3.53 (3.71–9.31) pg/mL vs. 11.05 (5.04–18.10) pg/mL, *p* = 0.037) (Figure 6A) (Table S7). Comparison of the paired samples showed that CXCL10 (*p* = 0.010) (Figure 6H), (Table S8) was significantly increased after treatment. Moreover, the change rate of IL‐6 (*p* = 0.036), IL‐8 (*p* = 0.004), CXCL10 (*p* = 0.001) (Figure 6C,F,I), IL‐1R1 (*p* = 0.036), IL‐9 (*p* = 0.027), FGF2 (*p* = 0.021), CSF3 (*p* = 0.0.036), CCR1 (*p* = 0.004), PDFGB (*p* = 0.027), CCL4 (*p* = 0.027), CCL5 (*p* = 0.001), and TNF (*p* = 0.006) was found to be significantly lower posttreatment in DCB patients than in NDB patients (Table S9).

##### 3.3.1.2 Survival analysis of ICI monotherapy cohort

Related to PFS and OS, univariate Cox regression showed that a high level of IL‐6 at pretreatment was associated with poor PFS (Table S10) and OS (Table S11). A high change rate of IL‐6, IL‐8, FGF2, CXCL10, CCR1, PDGFB, TNF, and APEX1 was consistently associated with poor PFS (Table S12). A high change rate of CXCL10 was the only factor to be significantly associated with poor OS (Table S13).

**FIGURE 7:**
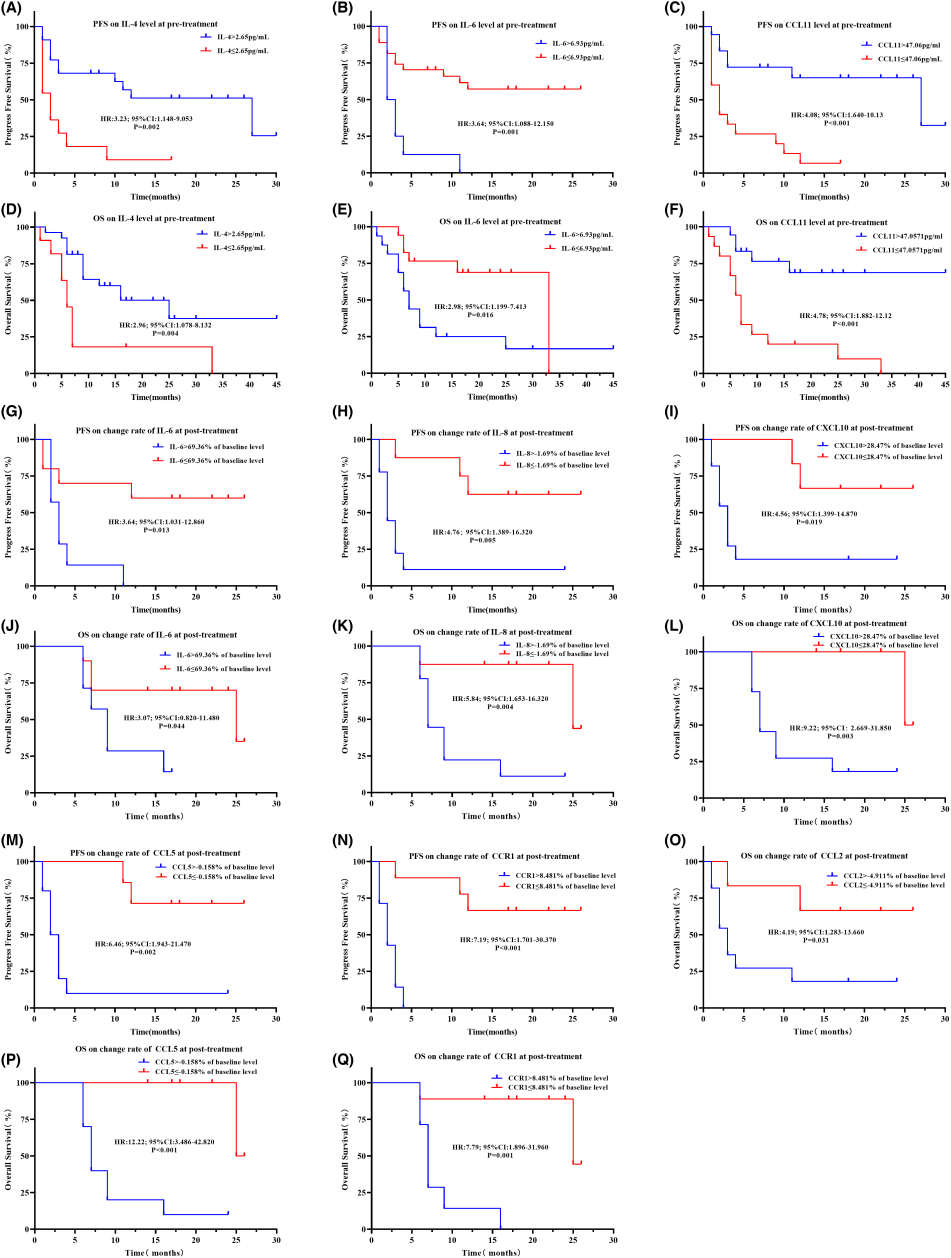
Kaplan–Meier curves illustrating differences in prognosis in the subset of patients receiving ICI monotherapy. (A) PFS based on IL‐4 level at pretreatment. (B) PFS based on IL‐6 level at pretreatment. (C) PFS based on CCL11 level at pretreatment. (D) OS based on IL‐4 level at pretreatment. (E) OS based on IL‐6 level at pretreatment. (F) OS based on CCL11 level at pretreatment. (G) PFS based on change rate of IL‐6. (H) PFS based on change rate of IL‐8. (I) PFS based on change rate of CXCL10. (J) OS based on change rate of IL‐6. (K) OS based on change rate of IL‐8. (L) OS based on change rate of CXCL10. (M) PFS based on change rate of CCL5. (N) PFS based on change rate of CCR1. (O) OS based on change rate of CCL2. (P) OS based on change rate of CCL5. (Q) OS based on change rate of CCR1.

The K–M curves showed that a low level of IL‐4, a high level of IL‐6, or a low level of CCL11 at pretreatment (Figure 7A–C), and a high change rate in IL‐6, IL‐8, CXCL10, CCL5, or CCR1 at posttreatment (Figure 7G–I, M,N), were significantly associated with poorer PFS. A low level of IL‐4 and a high level of IL‐6 at pretreatment (Figure 7D,E), or a high change rate of CCL11, IL‐6, IL‐8, CXCL10, CCL2, CCL5, or CCR1 (Figure 7J–L,O–Q) at posttreatment, were associated with poor OS as revealed by K–M curve analysis with assigned cut‐off values.

#### 3.3.2 Combination therapy cohort

##### 3.3.2.1 Characteristics of combinatory cohort

There were 18 patients that received combination therapy of ICI and chemotherapy. The median age of this cohort was 61 years (range, 31–83 years). The number of males and females was 14 (77.8%) and 4 (22.2%), respectively. Twelve (66.7%) patients were smokers. Among them, 13 (72.2%) and 5 (27.8%) were diagnosed with adenocarcinoma and squamous cell carcinoma. At the time of analysis, the median PFS and OS was 7.5 months (95% CI: 5.08–12.04) and 12 months (95% CI: 8.80–16.98), respectively. Eighteen or 12 plasma samples were collected at pretreatment or at 12 weeks posttreatment, respectively. According to RECIST1.1, there were 9 (50.0%), 5 (27.8%), and 4 (22.2%) participants evaluated as PR, SD, and PD, respectively. None of them was evaluated as CR.

A very different pattern of cytokine/chemokine alterations was observed in the combination therapy group with respect to the monotherapy group. In combination therapy, at pretreatment, FGF2, CCL4, and APEX1 levels were significantly lower in DCB patients than in NDB patients (Table S14). At 12 weeks posttreatment, IL‐4, IL‐7, IL‐12, IL‐17A, CSF3, CCR1, and TNF were significantly increased when compared with pretreatment (Table S15). After combination treatment, the change rate of IL‐4, IL‐13, FGF2, CCL2, PDFGB, and APEX1 was significantly lower in DCB patients than NDB patients (Table S16).

##### 3.3.2.2 Survival analysis of combination cohort

Cox regression showed that a high level of IL‐12, IL17‐A, FGF2, VEGF, and APEX1 at pretreatment and a high change rate of CCL2 were associated with poor PFS, whereas a high change rate of IL‐1R1 was associated with better PFS (Tables S17 and S18). High level of IL‐9, FGF2, PDFGB, CCL4, TFGB1, and APEX1 at pretreatment and high change rate of IL‐13, CSF2, and CCL2 were associated with poor OS (Tables S19 and S20). No independent factors were found for PFS or OS of the combination therapy group according to the multivariate Cox regression.

**FIGURE 8:**
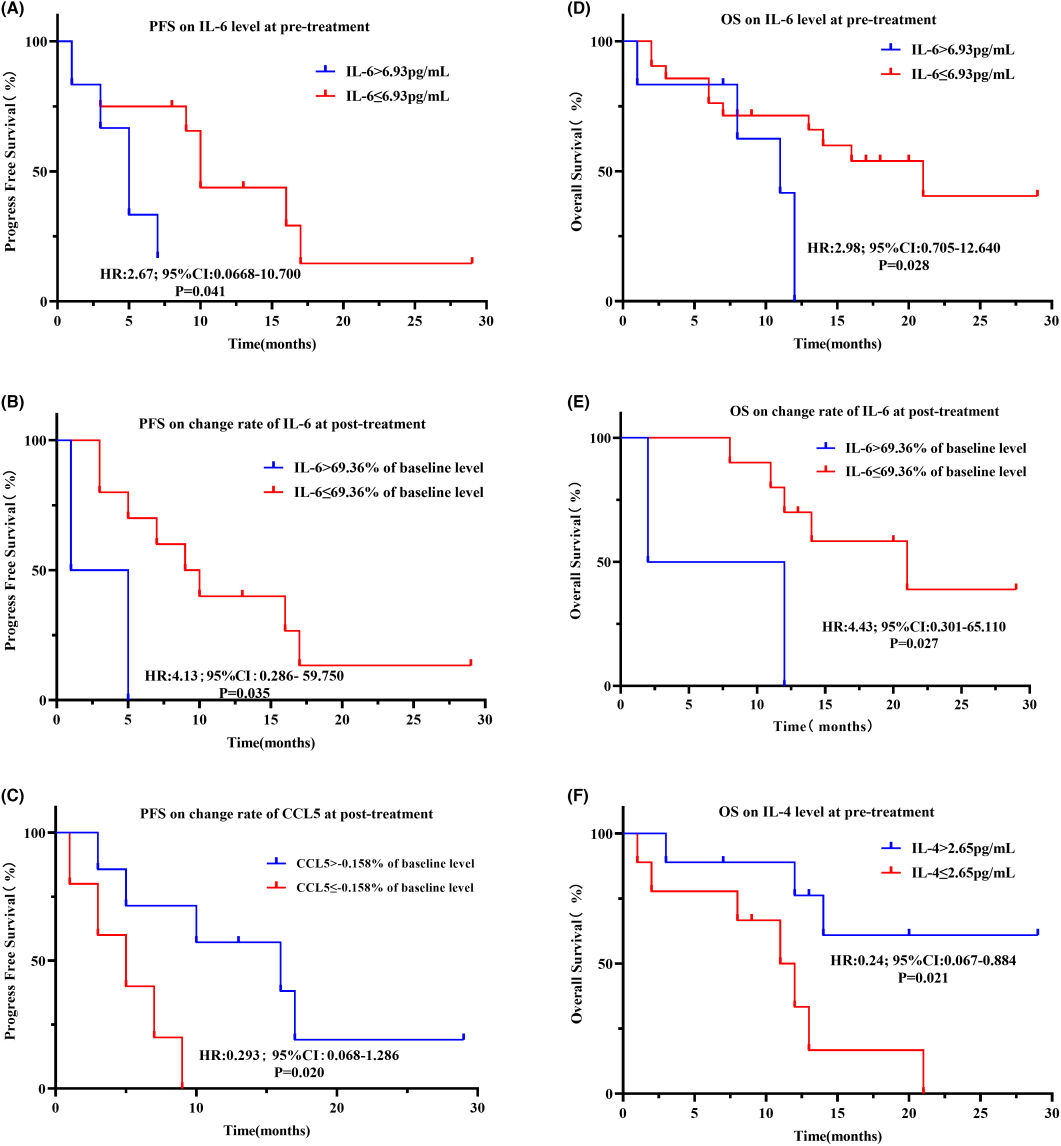
Kaplan–Meier curves illustrating differences in prognosis in the subset of patients receiving combination therapy. (A) PFS based on IL‐6 level at pretreatment. (B) PFS based on change rate of IL‐6. (C) PFS based on change rate of CCL5. (D) OS based on IL‐6 level at pretreatment. (E) OS based on change rates of IL‐6. (F) OS based on IL‐4 level at pretreatment.

The K–M curves were plotted using the same cut‐off values as defined above. In the combination therapy group, a high level of IL‐6 at pretreatment and a high change rate of IL‐6 or low change rate of CCL5 at posttreatment were associated with poor PFS (Figure 8A–C). A high level of IL‐6 at pretreatment and high change rate of IL‐6 at postreatment and a low level of IL‐4 at pretreatment were associated with shorter OS (Figure 8D–F). Compared with the ICI monotherapy cohort, patients with a higher change rate of CCL5 at posttreatment had a better PFS.

### 3.4 Distinct cytokine/chemokine profiles between ICI monotherapy and combination therapy

Cox regression analysis, following the introduction of cytokine levels and therapeutic regimens, further verified that several circulating factors differentially reflect prognosis based on either ICI monotherapy or combination therapy. The results show that PFS was differentially associated based on therapeutic regimen: change rate of IL‐8 (negative for ICI monotherapy, positive for ICI plus chemotherapy), CXCL10 (negative for ICI monotherapy, positive for ICI plus chemotherapy), CCL2 (negative for ICI monotherapy, positive for ICI plus chemotherapy), CCL5 (negative for ICI monotherapy, positive for ICI plus chemotherapy), or CCR1 (negative for both ICI monotherapy and ICI plus chemotherapy). The change rate of CCL5 (negative for ICI monotherapy, positive for ICI plus chemotherapy) exhibited a different impact on OS between the two therapeutic regimens (Table 8).

**TABLE 8:** Cox regression for progress free survival and overall survival with interaction of treatment regions and change rate of circulating factors.

		ICI monotherapy		ICI plus chemotherapy		*p* interaction
		*p*	HR (95% CI)	*p*	HR (95% CI)	
PFS	Change rate of IL‐8	0.028	2.820 (1.116–7.124)	0.655	0.746 (0.207–2.692)	0.011
	Change rate of CXCL10	0.042	5.143 (1.061–24.934)	0.795	0.843 (0.232–3.061)	0.048
	Change rate of CCL2^a^	0.060	4.482 (0.941–21.343)	0.145	0.261 (0.043–1.587)	0.037
	Change rate of CCL5	0.010	8.579 (1.684–43.718)	0.045	0.181 (0.034–0.959)	<0.001
	Change rate of CCR1	0.006	19.830 (2.351–167.238)	0.331	2.223 (0.444–11.130)	0.010
OS	Change rate of CCL5	0.109	86.350 (0.370–20125.281)	0.147	0.284 (0.052–1.554)	0.003

Abbreviation: ICI, immune checkpoint inhibitor.

^a^ Evaluated as continuous variate in the Cox regression and other factors as categorized variates (high vs. low) defined through the ROC analysis.

### 3.5 Association between circulating factors and ICI safety

In the PD‐1/PD‐L1 inhibitors monotherapy cohort (*n* = 33), we further explored the association between circulating factors and irAEs. According to CTCAE v5.0, a total of 19 irAEs events were observed (58% of the 33 total), with 7 (37%), 9 (47%), 2 (11%), and 1 (5%) being classified as grade 1, 2, 3, or 4, respectively. To exclude the survivor bias of toxicity, the time of irAEs occurrence was determined. There were 10 (53%), 3 (16%), 3 (16%), 1 (5%), and 2 (10%) out of the 19 irAEs that were recorded within 1, 2, 3, or 4 months or over 6 months, respectively. Skin toxicity was most frequently reported in our records (6 out of 19), with an increase in alanine transferase/aspartate transferase (ALT/AST) (5 out of 19) or a decrease in neutrophil count (4 out of 19) ranking second and third, respectively (Table S21).

We next explored the value of plasma cytokines in predicting the risk of irAEs. At both baseline (*n* = 33) and after treatment (*n* = 17), there were no significantly different cytokine levels between the patients with or without irAEs (Tables S22 and S23). However, patients with irAEs showed a longer OS than patients without irAEs (Figure S3).

## 4 DISCUSSION

In this study, we explored the correlation between the clinical response and the levels of a panel of peripheral cytokines and chemokines before and during treatment in a cohort of NSCLC patients receiving ICIs with or without chemotherapy. We observed a low level of IL‐4 at pretreatment, with IL‐4, IL‐12, IL‐17A, CSF3, TNF, and CCR1 being increased significantly posttreatment with ICIs. The analysis of paired samples showed that the level of CCL2, CCR1, and CCL5 changed more moderately posttreatment in DCB patients than in NDB patients. Additionally, distinct cytokine/chemokine profiles were observed in the ICI monotherapy and combination therapy cohorts. Specifically, in the monotherapy cohort, upregulation of FGF‐2, CSF3, CCR1, PDFGB, TNF, and APEX1 were associated with poor PFS. In the combination therapy cohort, a high level of IL‐12, IL‐17A, FGF2, VEGF, and APEX1 at pretreatment and a high change rate of IL‐1R1 were together associated with poor PFS. High levels of IL‐9, FGF2, PDFGB, CCL4, TFGB1, and APEX1 at pretreatment and a high change rate of IL‐13, CSF2, and CCL2 posttreatment were also associated with poor OS.

Taking into account the influence of the tumor‐intrinsic heterogeneity and of environmental factors on antitumor immunity, the relative change rate was used to normalize and assess the benefit after treatment. In the ICI monotherapy cohort, results revealed that a high change rate of IL‐6, IL‐8, CXCL10, CCL2, CCL5, and CCR1 were associated with poor PFS. Excluding CCL2, change rates in the other factors were also associated with poor OS. In the combination cohort, a high change rate for IL‐6 was associated with poor PFS and OS, while a high change rate of CCL5 was associated with better PFS; this latter observation is in contrast to the observation in the ICI monotherapy cohort. Our results therefore suggest that subsets of specific circulating cytokines and chemokines can potentially predict efficacy of ICIs in NSCLC patients, but with different patterns for ICI monotherapy and ICI combination therapy with platinum and taxol/gemcitabine/pemetrexed.

As the target of immunotherapy, the TME is consistently subjected to dramatic changes during ICI treatment. Thus, regular monitoring of these immunological changes is more important than just a baseline read of the TME. From this perspective, circulating immunological factor profiles provide a unique platform to evaluate and monitor clinical benefit of ICIs. Several previous studies have shed light on the relationship between some circulating factors and the clinical benefits of immunotherapy. But some studies have examined only individual circulating factors, while others lack the inclusion of ICI monotherapy. Previous analysis of peripheral factors suggested that the dysregulation of cytokines and chemokines can impact the clinical benefit of ICIs in the treatment of NSCLC.^28^, ^36^ For instance, a study showed that IL‐18 and CXCL10 are correlated with the degree of tumor response in 32 NSCLC patients who received PD‐1/PD‐L1 inhibitors. Moreover, patients with high CXCL10 expression displayed a shorter PFS than those with low CXCL10 expression.^28^ In another study, a decrease in the level of IL‐8 was associated with longer OS for melanoma or NSCLS patients treated with a PD‐1 inhibitor.^37^ Our results with ICI monotherapy and ICI combination therapy cohorts found similar results to these previous studies for IL‐8 and CXCL10.

Cytokines and chemokines play central roles in tumor angiogenesis, growth, and metastasis.^38^, ^39^, ^40^ Recent evidence indicates that malignant tumors generate an inflammatory microenvironment induced by the local release of cytokines and chemokines, making tumor‐infiltrating inflammation a hallmark of cancer.^41^ Dysfunction of cytokines and chemokines (e.g., IL‐1β, IL‐6, IL‐10, CCL2, CCL5) in TME of lung cancer were able to activate the tumor cell and inflammatory cell through signal pathway family such as nuclear factor‐kappa B (NF‐κB) family and signal transducer and activator of transcription (STAT) family which may lead to tumor immune escape, tumor angiogenesis, epithelial‐to‐transition (EMT) and anti‐apoptosis in lung cancer.^42^, ^43^, ^44^ In addition, CCL5 can decreased regulatory T cells (Tregs) through MAPK activation and promote an immune suppressive lung cancer environment.^45^ It was reported that under the stimulation of IL‐17A, the process of migration, invasion, and EMT can be promoted by NLRP3 activation in lung cancer.^46^ The role of IL‐17A in lung cancer treated with ICI is still not largely unknown, but a study showed that IL‐17A can increase the expression of PD‐L1 in colorectal cancer treated with anti‐PD‐1 therapy via p65/NRF1/miR‐15b‐5p and promotes the resistance of anti‐PD‐1 thearpy.^47^ Other work demonstrates that activation of certain cytokine/chemokine pathways, for example those involving IL‐6 or IL‐8, can promote the recruitment and proliferation of myeloid‐derived suppressor cells (MDSCs), which can contribute to the dysfunction of T cells in cancer immunity.^48^, ^49^, ^50^ Furthermore, the IL‐8, CXCL10, and CCR1 pathways can inhibit the function of cytotoxic T lymphocytes and NK cells leading to immune escape in anti‐PD‐1 and anti‐ CLTA‐4 mAb therapies.^51^, ^52^, ^53^ MDSCs and T regulatory cells are thought to be a component of immunogenic cell death (ICD), an association that could partly explain the role of cytokines/chemokines in ICI treatments.^54^, ^55^

We observed that an elevated level of APEX1, a key stress response enzyme that functions in DNA base excision repair, is associated with poor prognosis in both mono and combination ICI therapies. This finding suggests immune regulatory roles for APEX1 in NSCLC patients receiving ICIs, in addition to its established role in genotoxin chemotherapy responses. Defining the possible link between DNA repair and the immunotherapy response is an interesting avenue for future investigation.

As we observed a longer OS in patients with irAEs, irAEs might serve as an indicator of extended survival time during ISI treatment. This result is in at least partial agreement with other clinical studies.^56^, ^57^ However, given the lack of a patient report outcome system, the statistical power of irAEs could not be determined in the analysis here. Considering the current results, further studies seem warranted on the role of irAEs in predicting clinical outcomes for ICI therapies.

There are limitations to our study. First, there are several limitations from the nature of an exploratory study, the statistical power did not be determined planned comparisons to perform rigorous correction of multiple comparisons for all cytokines/chemokines studied. Second, due to losses during follow‐up and other majeure reasons, some paired samples were not collected. That, in particular, likely explains in part why some statistical significance was not achieved in the combination therapy cohort (see e.g., IL‐8 or CXCL10). Third, before accepting PD‐1/PD‐L1 inhibitor therapy, some patients were already undergoing chemotherapy. Even though subgroup analysis was performed, the effect of chemotherapy on cytokines/chemokines was therefore difficult to determine. Forth, some patients were not able to be tested for PD‐L1 status, and thus, we were unable to conduct full analysis of the impact of different PD‐L1 expression levels. Nevertheless, our results will serve as a basis for further investigations related to PD‐1/PD‐L1 inhibitor treatments.

Taken together, our study indicates that circulating cytokines/chemokines, which represent mildly noninvasive, easily accessible and reproducible indicators, might be valuable biomarkers in ICI treatments for lung cancer. Thus, we propose that different cytokine/chemokine profiles should be included in different ICI treatment strategies. Pretreatment cytokine/chemokine levels and continuous monitoring of level changes during treatment are both valuable for clinical efficacy assessment.

## AUTHOR CONTRIBUTIONS

**Yue Hu:** Data curation (supporting); formal analysis (supporting); investigation (supporting); writing – original draft (lead). **Shixun Li:** Data curation (lead); investigation (lead). **He Xiao:** Data curation (lead); formal analysis (equal); writing – original draft (supporting). **Yanli Xiong:** Resources (equal). **Xianfeng Lu:** Resources (equal). **Xiao Yang:** Resources (equal). **Wei Luo:** Resources (equal). **Jiamin Luo:** Resources (equal). **Shiheng Zhang:** Resources (equal). **Yi Cheng:** Resources (equal). **Lei Zhang:** Resources (equal). **Xiaoyan Dai:** Resources (equal). **Yuxin Yang:** Resources (equal). **Dong Wang:** Project administration (equal); supervision (equal). **Mengxia Li:** Funding acquisition (lead); methodology (lead); project administration (equal); resources (equal); supervision (equal); writing – review and editing (lead).

## FUNDING INFORMATION

This study was supported by the Chongqing Science Fund for Distinguished Young Scholars (cstc2019jcyjjqX0008) and Science and Technology Innovation Enhancement Project of Army Medical University (2019XLC3057).

## CONFLICT OF INTEREST STATEMENT

The authors have no conflict of interest to declare.

## ETHICS STATEMENT

This study was approved by the Ethics Committee of Daping Hospital [2022(105)].

## Supporting information


**Supplementary Figure 1** Line series showing dynamics before and after ICI treatment in IL‐4 **(a)**，IL‐12 **(b)**, IL‐17A **(c)**, CSF3 **(d)**, CCR1 **(e)** and TNF **(f)** of full cohort.

**Supplementary Figure 2** Box plot showing difference in change rate of CCL2 **(a)**, CCL5 **(b)** and CCR1 **(c)** between NDB and DCB patients of complete cohort.

**Supplementary Figure 3** Kaplan–Meier curves illustrating difference in prognosis on the occurrence of irAEs.

Table S1:

## Data Availability

Data sharing is not applicable to this article as no new data were created or analyzed in this study.
