# Supplementary figures and images for: Distinct circulating cytokine/chemokine profiles correlate with clinical benefit of immune checkpoint inhibitor monotherapy and combination therapy in advanced non‐small cell lung cancer
Source: Cancer Med. 2023 Apr 16;12(11):12234–52. doi: 10.1002/cam4.5918 (PMC10278479; doi:10.1002/cam4.5918)

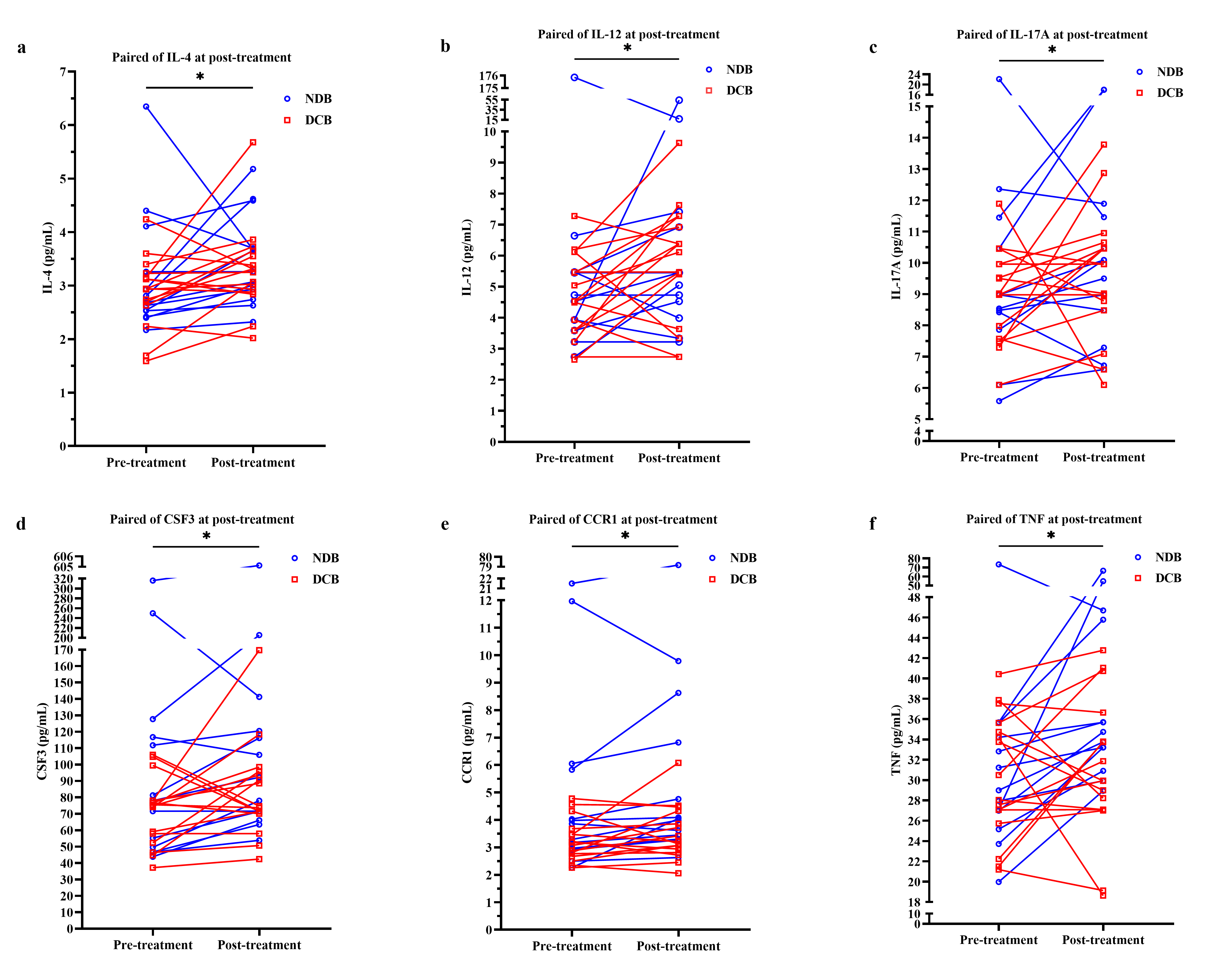

Supplement: Supplementary file 1 — Supplementary Figure 1 Line series showing dynamics before and after ICI treatment in IL‐4 (a)，IL‐12 (b), IL‐17A (c), CSF3 (d), CCR1 (e) and TNF (f) of full cohort. [file CAM4-12-12234-s004.tif]

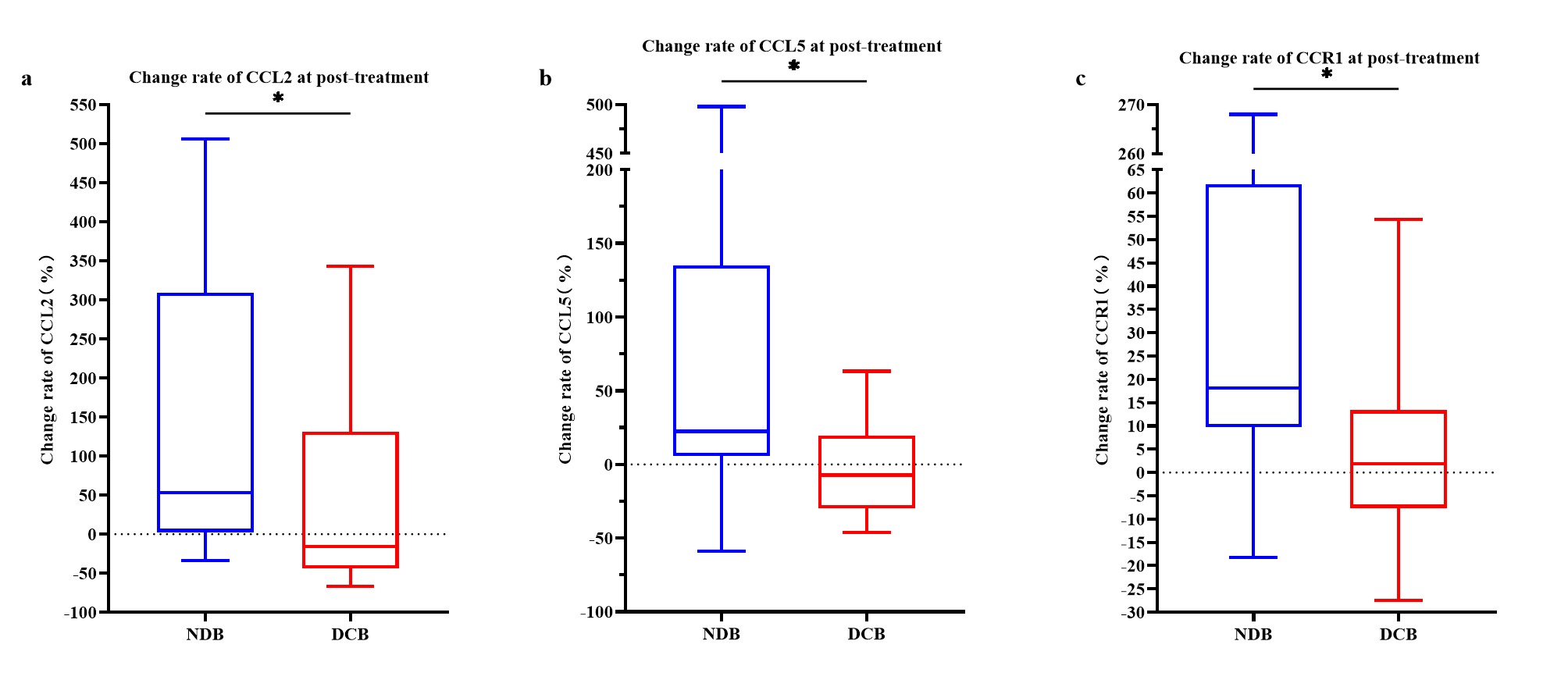

Supplement: Supplementary file 2 — Supplementary Figure 2 Box plot showing difference in change rate of CCL2 (a), CCL5 (b) and CCR1 (c) between NDB and DCB patients of complete cohort. [file CAM4-12-12234-s001.tif]

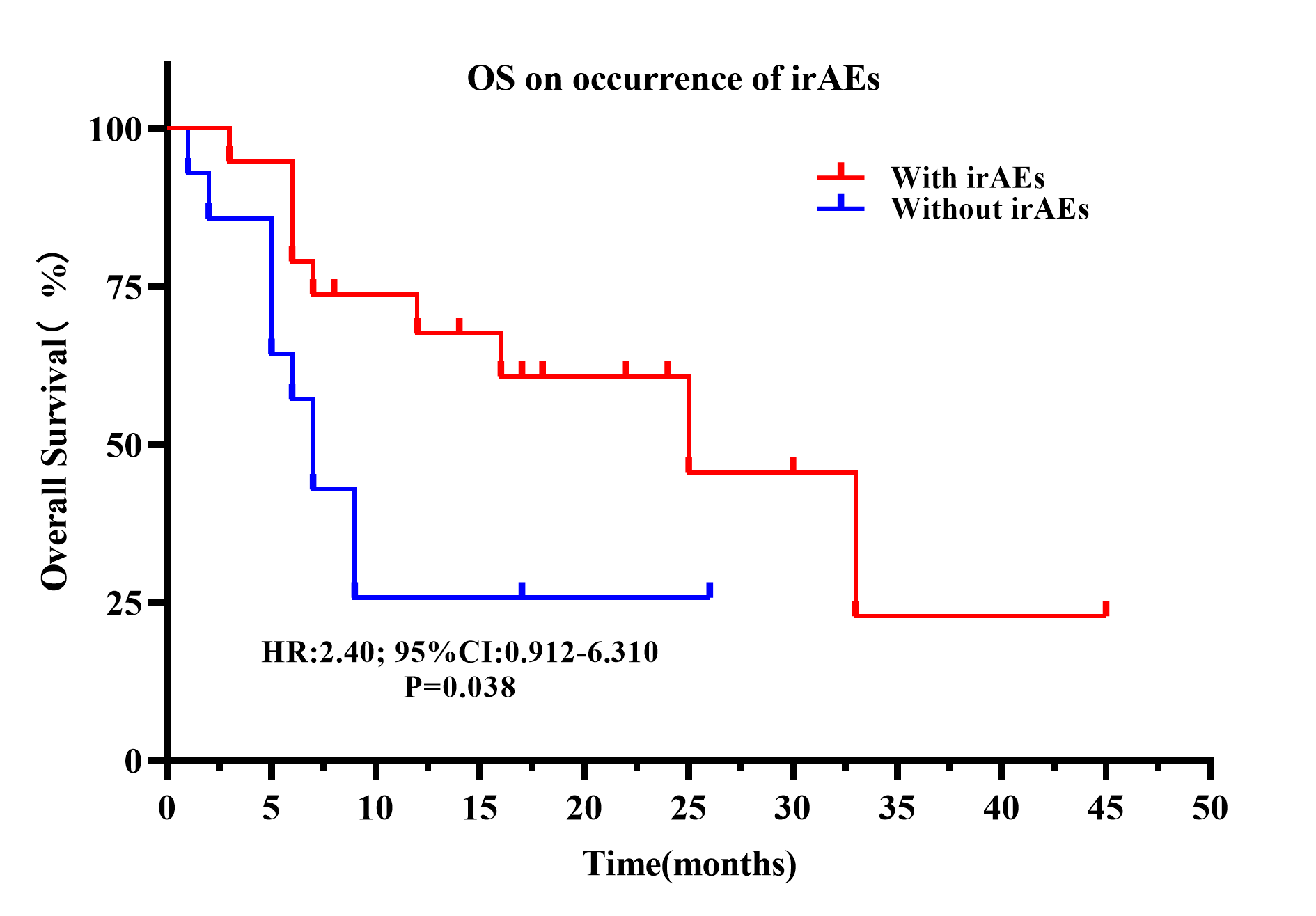

Supplement: Supplementary file 3 — Supplementary Figure 3 Kaplan–Meier curves illustrating difference in prognosis on the occurrence of irAEs. [file CAM4-12-12234-s002.tif]
